# Increasing Knowledge and Health Literacy about Preterm Births in Underserved Communities: An Approach to Decrease Health Disparities, a Pilot Study

**DOI:** 10.5539/gjhs.v8n1p83

**Published:** 2015-05-15

**Authors:** Allison A. Vanderbilt, Marcie S. Wright, Alisa E. Brewer, Lydia K. Murithi, PonJola Coney

**Affiliations:** 1 Center on Health Disparities, Assistant Professor, School of Medicine, Virginia Commonwealth University, Richmond, Virginia, USA

**Keywords:** health disparities, underserved communities, public health, preterm birth, community based participatory research, prevention, tailored intervention

## Abstract

**Introduction:**

Health disparities can negatively impact subsets of the population who have systematically experienced greater socioeconomic obstacles to health. For example, health disparities between ethnic and racial groups continue to grow due to the widening gap in large declines in infant and fetal mortality among Caucasians compared to Black non-Hispanic or African Americans. According to the American Congress of Obstetricians and Gynecologists, preterm birth remains a leading cause of infant morbidity and mortality. The purpose of our study is to determine if the computer-based educational modules related to preterm birth health literacy and health disparity with a pre-test and post-test can effectively increase health knowledge of our participants in targeted underserved communities within the Richmond-metro area.

**Methods:**

This was a pilot study in the Richmond-Metro area. Participants were required to be over the age of 18, and had to electronically give consent. Descriptive statistics, means and standard deviations, and Paired *t*-tests were conducted in SPSS 22.0.

**Results:**

There were 140 participants in the pilot study. *P* <.05 was set as significant and all four modules had a *P* <.000. The males were *not* significant with modules: Let’s Talk Patient & Provider Communication *P* <.132 and It Takes a Village *P* <.066. Preterm birth status yes all of the findings were statistically significant *P*<.000. Preterm birth status no Let’s Talk Patients & Provider Communication was not significant *P* <.106.

**Conclusion:**

Overall, researchers found that with a strong research methodology and strong content relevant to the community, the participants demonstrated an increase in their knowledge in health literacy and preterm birth.

## 1. Introduction

Health disparities can negatively impact subsets of the population who have systematically experienced greater socioeconomic obstacles to health (Vanderbilt, Dail, & Jaberi, 2015). For example, health disparities between ethnic and racial groups continue to grow (Heron, Sutton, Xu, Ventura, Strobino, & Guyer, 2009) due to the widening gap in large declines in infant and fetal mortality among Caucasians compared to Black non-Hispanic or African Americans (Wingate, Barfield, Petrini, & Smith, 2012). According to the American Congress of Obstetricians and Gynecologists, preterm birth remains a leading cause of infant morbidity and mortality (The American Congress of Obstetricians and Gynecologists: 2011 Women’s Health Stats & Facts). With this influx of infant mortality across the United States, it is imperative to incorporate health disparities which affect infant mortality and high risk pregnancies (Vanderbilt & Wright, 2013) in ways that decrease these negative outcomes in underserved communities.

Unfortunately, in the United States, Black non-Hispanic and African American women give birth to preterm babies more frequently than the rest of women in United States, and have a 50% greater infant mortality and still birth rate (Spong, Iams, Goldenberg, Hauack, & Willinger, 2011). The Center for Disease Control and Prevention (CDC) proclaim it is essential to increase community awareness of disparities because these are some of the most persistent problems and serious health challenges within United States (CDC.gov, http://www.cdc.gov/minorityhealth/CHDIR/2011/FactSheets/PretermBirth.pdf); such as, preterm births. Therefore, the purpose of our study is to determine if the computer-based educational modules related to preterm birth health literacy and health disparity with a pre-test and post-test can effectively increase health knowledge of our participants in targeted underserved communities within the Richmond-metro area.

## 2. Methods

The intervention focused on five computer-based educational modules. The computers used in this study were laptops Dell Latitude E6430. The first module assessed computer literacy. It was critical to determine that participants had or could achieve basic computer skills scores of 70% of higher, since the intervention was computer based. All of the modules were assessed for readability to assure that the content did not exceed a fourth grade reading level. To further reduce literacy barriers, each participant was provided with their own set of earphones so they were able to listen to the narrated audio recording as they read along.

To boost health literacy and health disparity education, content for all modules was developed with help and direction from experts in obstetrics and gynecology, health literacy, instructional development, and input from community partners (e.g., intervention site staff, their patrons and neighbors, as well as community health initiative representatives). This guidance directed material for each module to assure the material was current and relevant for learning purposes in the target population. Content was formatted for delivery using online storyboard training software (Articulāte) for an attractive, user-friendly experience. Module activity was interactive and allowed for participants to have engaged/active learning opportunities; in other words, adult learning theory was employed to meet the various learning style needs of the learners, integrating computer-based active learning such as game-like quizzes and community-relatable avatars to deliver the health messages. Topic-specific and interactive “Health Disparity Alerts” were featured per module to illustrate in plain language how health disparities impact population health at local and national levels. Finally, an online repository resource of Internet links for continued learning and community support services followed each module. The following briefly describes the content for the five modules as recommended by community input for preterm birth prevention literacy, health disparity education, and overall health:

1) **Computer Basics: The crash course to computer use and getting around this training resource** teaches the first steps to using computers with practice activities.
2) **Born Too Soon: The peer health advocate’s toolkit to preterm birth** teaches what preterm birth is, why it is a problem, and behavior practices to solve the problem.
3) **The Internet and My Health: How and where to search online** teaches the best places to search online, as well as how to self-evaluate online information for accuracy and reliability.
4) **Let’s Talk: Support for patient and provider communication** teaches participants health literacy and health communication skills, for patients and providers.
5) **It Takes a Village: Life skills for community peer health leaders** reviews life skills for community health peer leaders. Content addresses community advocacy, civic engagement, and job readiness.

Before taking part in computer-based educational modules, the software system guided participants to create a login username and password, to submit informed consent, and complete a demographic survey. Module activity included a pre-test containing 10 item multiple-choice questions that were content related to the module they were going to learn about – introduced as, “to see what participants already know about the topic”. Following the module a post-test was administered with 10 item multiple-choice questions – “to see what participants have learned”. The participants were allowed to pace themselves and could return at a later time to finish intervention activity. Therefore, the time between the pre-test and post-test varied for each participant since it was individual and they were allowed to pace themselves. However, each participant was given an hour to use the computer; but if they needed more time they were allowed to exceed that. The first author is not aware of any participant requesting additional time beyond an hour. Readability was also taken into consideration for all of the pre-test and post-test questions that were administered. They did not exceed fourth grade level to assure that the participants would be able to read and understand the questions being asked of them. The goal was to measure their learning of knowledge not how well they could read.

Approval from the Virginia Commonwealth University Institutional Review Board was obtained September 2014 prior to starting the pilot study. Participants were required to be over the age of 18, pass the computer skills test with a 70% or higher, live in the Richmond-Metro area community serviced by one of the resource centers, and submit consent in order to be involved with the pilot study. Additionally, the study was conducted on site in the resource centers in the participants’ community. Descriptive statistics, means and standard deviations, and paired *t*-tests were conducted in SPSS 22.0. Our analysis was used to addresses our hypothesis/purpose of our study previously stated.

## 3. Results

There were 140 participants in the pilot study. Table 1 provides the demographics of the participants. There were 20% males and 80% females that elected to be involved with the pilot study and 84% were single. In addition it is noteworthy that 95% of the participants were Black or African American and 75% made less than #10,000 annually. Finally, 39% of the participants reported that they were unemployed and not looking for work and 46% completed the 12^th^ grade or less.

**Table 1:** Demographics of Participants

Characteristic	(N = 140)
Gender, %	
Male	20.0
Female	80.0
Household Income, %	
>10,000	75.0
10,000-19,999	18.0
20,000-29,999	5.0
30,000-39,999	1.0
40,000-49,999	0.7
Marital Status	
Single	84.0
Married	7.0
Widowed	0.7
Separated	4.0
Divorced	4.0
Race/Ethnicity, %	
Black or African-American	95.0
American Indian or White or Caucasian or Hispanic or Latino Black or Hispanic	0.7
White or Caucasian	0.7
Asian or Pacific Islander	1.0
American Indian or Black or African-American	1.0
American Indian	0.7
Education Level, %	
High School Degree	32.0
12^th^ Grade or Less	46.0
Some College	13.0
Associate’s Degree or Technical Program	6.0
Bachelor’s Degree	3.0
Graduate or Professional Degree	0.7
Employment Status, %	
Employed Part-time	19.0
Employed Full-time	15.0
Unemployed & Looking	7.0
Unemployed & Not Looking	39.0
Unable to Work / Disability	18.0
Student	3.0

*Note*. Percentages may not total because of rounding.

In Table 2, the paired samples *t*-test by module and *P* value is reported out. It is noteworthy that all four modules (1) Born too soon; (2) My Health & The Internet; (3) Let’s Talk Patient & Provider Communication; and (4) It Takes a Village were all found to be statistically significant. *P <*.05 was set as significant and all four modules had a *P* <.000. Table 3 demonstrates the mean and standard deviations for these modules.

**Table 2:** Paired Samples *T*-Test by Module

Module	*P* Value
**Born Too Soon**	*p* <.000*
**My Health & The Internet**	*p* <.000*
**Let’s Talk Patient & Provider Communication**	*p* <.000*
**It Takes A Village**	*p* <.000*

* *Note.* N=140, *p* < .05 = significant.

**Table 3:** Aggregate Means and Standard Deviations by Module

Module	Mean ± SD
**Born Too Soon PreTest**	80.36 ± 20.40
**Born Too Soon PostTest**	90.93 ± 13.67
**My Health & The Internet PreTest**	83.36 ± 22.77
**My Health & The Internet PostTest**	90.36 ± 15.75
**Let’s Talk Patient & Provider Communication PreTest**	84.64 ± 22.83
**Let’s Talk Patient & Provider Communication PostTest**	91.71 ± 16.81
**It Takes a Village PreTest**	67.23 ± 20.76
**It Takes a Village PostTest**	75.67 ± 15.73

*Note*. *SD* = standard deviation, N=140.

There was an interesting finding with Table 4, the paired samples *t*-test by gender and by module. The females in all four modules were significant at *P* <.000; however, the males varied. The males were *not* significant with: Let’s Talk Patient & Provider Communication *P* <.132 and It Takes a Village *P* <.066. The means and standard deviations for the females and males can be found in Table 5.

**Table 4:** Paired Samples *T*-Test by Module and by Gender

Module	Male	Female
**Born Too Soon**	*p* <.000*	*p* <.000*
**My Health & The Internet**	*p* <.001*	*p* <.000*
**Let’s Talk Patient & Provider Communication**	*p* <.132	*p* <.000*
**It Takes A Village**	*p* <.066	*p* <.000*

* *Note.* N=140, p < .05 = significant.

**Table 5:** Means and Standard Deviations by Module by Gender

Module	Mean ± SD
**Born Too Soon PreTest (Male)**	71.07 ± 26.01
**Born Too Soon PostTest (Male)**	88.57 ± 13.25
**My Health & The Internet PreTest (Male)**	80.71 ± 23.24
**My Health & The Internet PostTest (Male)**	88.57 ± 15.08
**Let’s Talk Patient & Provider Communication PreTest (Male)**	78.57 ± 26.76
**Let’s Talk Patient & Provider Communication PostTest (Male)**	85.71 ± 15.73
**It Takes a Village PreTest (Male)**	63.21 ± 20.73
**It Takes a Village PostTest (Male)**	75.71 ± 15.49
**Born Too Soon PreTest (Female)**	82.68 ± 18.15
**Born Too Soon PostTest (Female)**	91.52 ± 13.78
**My Health & The Internet PreTest (Female)**	84.02 ± 22.71
**My Health & The Internet PostTest (Female)**	90.80 ± 15.94
**Let’s Talk Patient & Provider Communication PreTest (Female)**	86.16 ± 21.61
**Let’s Talk Patient & Provider Communication PostTest (Female)**	93.21 ± 16.00
**It Takes a Village PreTest (Female)**	68.21 ± 20.76
**It Takes a Village PostTest (Female)**	75.54 ± 15.87

*Note*. *SD* = standard deviation, N=140.

Finally, there was another interesting finding with Table 6, paired samples *t*-test by modules and previous preterm birth status. For the preterm birth status *yes* all of the findings were statistically significant *P*<.000; however, with the preterm birth status *no*, they were not all significant. The module, Let’s Talk Patients & Provider Communication was not significant *P*<.106. For the mean and standard deviations for this section refer to Table 7.

**Table 6:** Paired Samples *T*-Test by Module & by Previous Preterm Birth Status

Module	Yes Preterm Birth	No Preterm Birth
**Born Too Soon**	*p* <.000*	*p* <.002*
**My Health & The Internet**	*p* <.000*	*p* <.021*
**Let’s Talk Patient & Provider Communication**	*p* <.000*	*p* <.106
**It Takes A Village**	*p* <.000*	*p* <.005*

* *Note.* N=140, *p* < .05 = significant.

**Table 7:** Means and Standard Deviations by Module by Previous Preterm Birth Status

Module	Mean ± SD
**Born Too Soon PreTest (Yes)**	82.96 ± 18.23
**Born Too Soon PostTest (Yes)**	93.16 ± 11.71
**My Health & The Internet PreTest (Yes)**	84.69 ± 22.02
**My Health & The Internet PostTest (Yes)**	91.73 ± 13.77
**Let’s Talk Patient & Provider Communication PreTest (Yes)**	86.63 ± 21.24
**Let’s Talk Patient & Provider Communication PostTest (Yes)**	94.49 ± 14.29
**It Takes a Village PreTest (Yes)**	67.04 ± 21.73
**It Takes a Village PostTest (Yes)**	75.61 ± 16.37
**Born Too Soon PreTest (No)**	82.68 ± 16.40
**Born Too Soon PostTest (No)**	74.29 ± 23.90
**My Health & The Internet PreTest (No)**	80.24 ± 24.44
**My Health & The Internet PostTest (No)**	87.14 ± 19.41
**Let’s Talk Patient & Provider Communication PreTest (No)**	80.00 ± 25.85
**Let’s Talk Patient & Provider Communication PostTest (No)**	85.24 ± 18.51
**It Takes a Village PreTest (No)**	67.62 ± 18.58
**It Takes a Village PostTest (No)**	75.48 ± 14.34

*Note*. *SD* = standard deviation, N=140.

## 4. Limitations

The participants were only from one geographical radius surrounding the Richmond-Metro area in Virginia. It is not evident how this material would be applicable to a larger sample such as a rural community or across other regions within the United States. Additional research needs to be conducted to determine how this series of modules focused on preterm birth and health literacy can benefit other communities and other populations. A larger sample size would be beneficial. The participants were not followed up with to check on knowledge retention–an interesting study would be to determine if they retained what they learned six months later.

## 5. Discussion

The findings in this pilot study are strong to suggest that the computer-based modules developed (intervention) to educate adults in an underserved community about preterm birth and health literacy has the potential to decrease health disparities related to this area. The researchers believe these findings were strong due to several key factors: (1) methodology; (2) computer and Internet skills; and (3) readability.

Although, this was a pilot study, it was imperative that the methods to this study be strong in order to measure the data accurately with validity and reliability. Therefore, having a unique login for each participant assured that they were matched to their pre and post test scores only. It was critical to be specific with who could participate in the research study by the computer and not by a person; thus it eliminated human error, and allowed for each participant to be their own individual control therefore we could measure their growth and learning based on the modules. Secondly, the researchers required that participants have basic computer skills to participate in the pilot study. We were not studying nor measuring how people perform on a computer, therefore we didn’t want that to be a variable, we wanted to learn can individuals increase their knowledge about preterm birth and health literacy based on the modules. Having a cut off score of 70% allowed us to eliminate participants who were not computer savvy enough to actively engage in the modules. Finally, having a readability level of fourth grade met the level of our participants. All participants were given their own earplugs to listen to the voice recording as well so they could read along as they listened; however, it was essential that the modules, pre and post tests be written using vocabulary that would be at a level readable to fourth graders. This ensured that all participants would feel comfortable reading as well as reach the target audience for educating them about preterm birth and health literacy. We wanted to assure that the material would be appropriate for learning especially since we knew the majority of our target demographics were in underserved communities and most likely would not have completed high school.

Overall, the researchers found that with a strong research methodology and strong content relevant to the community, the participants demonstrated an increase in their knowledge in health literacy and preterm birth.
